# Correction: Type III CRISPR-Cas systems can provide redundancy to counteract viral escape from type I systems

**DOI:** 10.7554/eLife.36853

**Published:** 2018-04-04

**Authors:** Sukrit Silas, Patricia Lucas-Elio, Simon A Jackson, Alejandra Aroca-Crevillén, Loren L Hansen, Peter C Fineran, Andrew Z Fire, Antonio Sánchez-Amat

Silas S, Lucas-Elio P, Jackson SA, Aroca-Crevillén A, Hansen LL, Fineran PC, Fire AZ, Sánchez-Amat A. 2017. Type III CRISPR-Cas systems can provide redundancy to counteract viral escape from type I systems. *eLife* **6**:e27601. doi: 10.7554/eLife.27601.

Published 17, August 2017

A typographical error on our part was identified in Figure 6B. We erroneously labeled one cmr6 gene in the type III-B CRISPR-Cas system in *M. mediterranea* CPR1 as a cas6 instead.

The corrected figure 6 is shown here:

**Figure:**
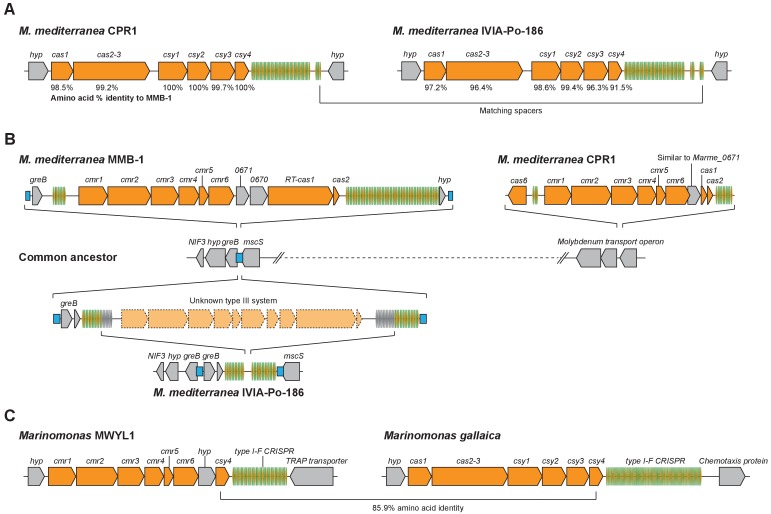


The originally published figure 6 is shown for reference:

**Figure:**
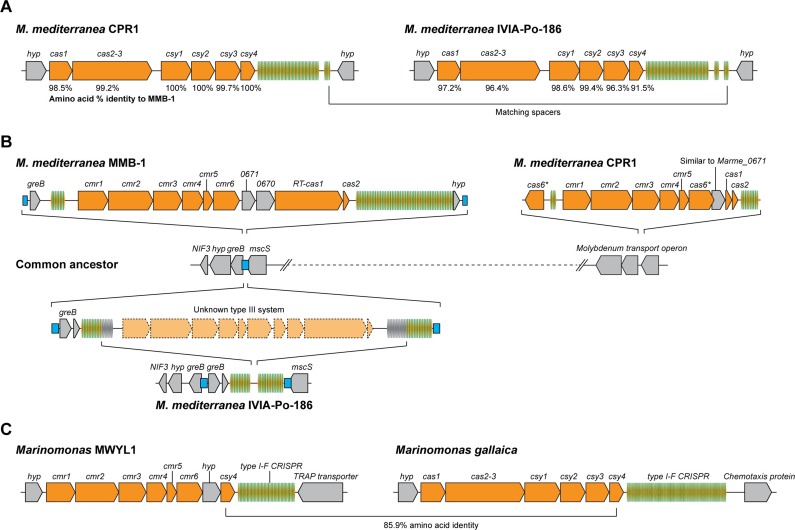


The language in the following sentence of the Discussion describing this type III-B CRISPR-Cas system has been amended to account for this correction.

Original text:

In addition, the ability to process type I pre-crRNAs might be carried within some type III operons; for example, the type III-B system in *M. mediterranea* CPR1 encodes two diverse Cas6-family proteins (Figure 6) that may enable the system to independently process pre-crRNAs from different CRISPR-Cas systems.

Corrected text:

In addition, the ability to process type I pre-crRNAs might be carried within some type III systems; for example, the type III-B system in *M. mediterranea* CPR1 encodes a divergent Cas6-family protein (Figure 6) that may enable the system to independently process pre-crRNAs from different CRISPR-Cas systems.

The article has been corrected accordingly.

